# Guidelines for Feeding Very Low Birth Weight Infants

**DOI:** 10.3390/nu7010423

**Published:** 2015-01-08

**Authors:** Sourabh Dutta, Balpreet Singh, Lorraine Chessell, Jennifer Wilson, Marianne Janes, Kimberley McDonald, Shaneela Shahid, Victoria A. Gardner, Aune Hjartarson, Margaret Purcha, Jennifer Watson, Chris de Boer, Barbara Gaal, Christoph Fusch

**Affiliations:** Division of Neonatology, Department of Pediatrics, McMaster University Children’s Hospital, Hamilton L8S4L8, Ontario, Canada; E-Mails: drbalpreetsingh@yahoo.com (B.S.); chessell@HHSC.CA (L.C.); wilsonj@HHSC.CA (J.W.); janes@HHSC.CA (M.J.); mcdonk@HHSC.CA (K.M.); shahidsattar75@yahoo.com (S.S.); gardner@hhsc.ca (V.A.G.); hjartar@hhsc.ca (A.H.); purcham@HHSC.CA (M.P.); watsonje@hhsc.ca (J.W.); deboerc@hhsc.ca (C.B.); B.Gaal@bell.net (B.G.); fusch@mcmaster.ca (C.F.)

**Keywords:** feeding, very low birth weight, neonate, review

## Abstract

Despite the fact that feeding a very low birth weight (VLBW) neonate is a fundamental and inevitable part of its management, this is a field which is beset with controversies. Optimal nutrition improves growth and neurological outcomes, and reduces the incidence of sepsis and possibly even retinopathy of prematurity. There is a great deal of heterogeneity of practice among neonatologists and pediatricians regarding feeding VLBW infants. A working group on feeding guidelines for VLBW infants was constituted in McMaster University, Canada. The group listed a number of important questions that had to be answered with respect to feeding VLBW infants, systematically reviewed the literature, critically appraised the level of evidence, and generated a comprehensive set of guidelines. These guidelines form the basis of this state-of-art review. The review touches upon trophic feeding, nutritional feeding, fortification, feeding in special circumstances, assessment of feed tolerance, and management of gastric residuals, gastro-esophageal reflux, and glycerin enemas.

## 1. Introduction

Adequate nutrition is essential for the optimal growth and health of very low birth weight (VLBW) infants. Enteral nutrition is preferred to total parenteral nutrition (TPN) because the former avoids complications related to vascular catheterization, sepsis, adverse effects of TPN, and fasting. Early parenteral nutrition in these babies remains critical and should be used as an adjunct to enteral nutrition. The overarching goal while feeding VLBW infants (VLBWI) is to reach full enteral feeding in the shortest time, while maintaining optimal growth and nutrition and avoiding the adverse consequences of rapid advancement of feeding. Attaining this goal is more difficult than it sounds, and controversies abound.

A multi-disciplinary working group in McMaster University (comprised of staff neonatologists, fellows, nutritionists, nurse practitioners, nurses, lactation consultants, and occupational therapists) conducted a structured literature search, critically appraised the evidence, presented it to a wider group of neonatologists, and came up with practical suggestions to feed VLBWI—the basis for this review. There are some areas where there is limited evidence, and in these areas we have suggested reasonable approaches based on expert consensus. Wherever possible, we have stated the level of evidence (LOE) as per the Centre for Evidence-based Medicine, United Kingdom [1]. The outline of the LOE for therapy trials is as follows:

1a Systematic review (with homogeneity) of randomized controlled trials (RCT)
1b Individual RCT with narrow confidence interval (CI)
2a Systematic review (with homogeneity) of cohort studies
2b Individual cohort studies and low-quality RCTs
3a Systematic review (with homogeneity) of case-control studies
3b Individual case-control studies
4 Case series, poor-quality cohort and poor-quality case-control studies
5 Expert opinion without explicit critical appraisal

If a minus sign is suffixed (e.g., 1a− or 1b−), it denotes either a single study with wide CI or a systematic review with troublesome heterogeneity.

## 2. Time to Reach Full Feeds

### 2.1. Suggestion

Aim to reach full enteral feeding (~150–180 mL/kg/day) by about two weeks in babies weighing <1000 g at birth and by about one week in babies weighing 1000–1500 g by implementing evidence-based feeding protocols. It may be noted that some babies, especially those less than 1000 grams, will not tolerate larger volumes of feedings (such as 180 mL/kg/day or more) and thus may need individualization.

### 2.2. Rationale

Reaching full enteral feeding faster results in earlier removal of vascular catheters, and less sepsis and other catheter-related complications (LOE 2b) [2,3,4]. Standardized feeding protocols improve outcomes in VLBWI [4,5]. Reaching full feeds within a week is achievable—in an RCT on VLBWI, the median time to reach 170 mL/kg/day was 7 days after fast advancement of enteral feeding, with no increase in apneas, feed interruptions, and intolerance [6].

## 3. Frequency of Feeds

### 3.1. Suggestion

Administer three-hourly feeds for babies weighing >1250 g. There is not enough evidence to choose between two-hourly *versus* three-hourly feeds for babies weighing ≤1250 g.

### 3.2. Rationale

In an RCT, 92 neonates weighing <1750 g were allocated to either three- or two-hourly feeds [7]. The incidence of feed intolerance, apnea, hypoglycemia, and necrotizing enterocolitis (NEC) did not significantly differ, and nursing time spent on feeding was significantly less in the three-hourly group (LOE 2b).

Two retrospective studies on this issue were contradictory. In one that compared 2-h and 3-h enteral feeding in ELBW babies, the time to full enteral feeding, enteral morbidity, hospital stay, and growth parameters were similar in the two groups (LOE 4) [8]. In another, VLBWI (mean birth weight ~1200 g) fed twice hourly reached full feeds faster, received less prolonged TPN, and were less likely to have feeds held, compared to those fed three times hourly (LOE 4) [9]. Putting this limited information together, we propose that babies weighing ≥1250 g be fed three times hourly and those weighing <1250 g preferably twice hourly.

## 4. Trophic Feeds: Time of Starting, Volume, Duration

### 4.1. Suggestion

Trophic feeds are defined as minimal volumes of milk feeds (10–15 mL/kg/day). Start trophic feeds preferably within 24 h of life. Exercise caution in extremely preterm, extremely low birth weight (ELBW), or growth-restricted infants. If, by 24–48 h, no maternal or donor milk is available, consider formula milk. There is not enough evidence to recommend the maximum duration of trophic feeding before starting nutritional feeds.

### 4.2. Rationale

In a systematic review (nine trials, 754 VLBWI), the actual volume of trophic feeds ranged from 10 to 25 mL/kg/day; and onset from day one of life onwards [10]. Early introduction of trophic feeds compared to fasting had a non-significant trend towards reaching full feeds earlier (mean difference − 1.05 days (95% CI −2.61, 0.51)) and no difference in NEC (LOE 1a−). More data is required before one can generalize these findings to extremely preterm, ELBW, or growth-restricted infants.

There was no subgroup analysis on formula milk. Among the included studies, there were two studies in which trophic feeding was provided exclusively by preterm formula (LOE 1b−) [11,12]. In both, the trophic feeding group had less feeding intolerance and reached full feeds faster without increase in NEC. Hence, formula milk may be used after exhausting other options. We suggest a reasonable waiting period of 24–48 h for obtaining maternal or donor milk.

In a systematic review (seven trials, 964 VLBWI) on timing of introduction of nutritional enteral feeding to prevent NEC, early introduction of progressive enteral feeding (1 to 2 days of age) did not increase the risk of NEC (typical relative risk (RR) 0.92 (95% CI 0.64, 1.34)), mortality (typical RR 1.26 (95% CI 0.78, 2.01)), or feed intolerance (LOE 1a) [13]. We converted this into a practical suggestion of the maximum number of days for trophic feeding before introducing progressive enteral feeding.

## 5. Contraindications for Trophic Feeds

### 5.1. Suggestion

Withhold trophic feeds in intestinal obstruction or a setting for intestinal obstruction or ileus.

Asphyxia, respiratory distress, sepsis, hypotension, glucose disturbances, ventilation, and umbilical lines are not contraindications for trophic feeds.

### 5.2. Rationale

The studies included in a Cochrane review included VLBWI with asphyxia, respiratory distress, sepsis, hypotension, glucose disturbances, ventilation, and umbilical lines, without any excess adverse effects being reported (LOE 1a−) [10].

## 6. Nutritional Feeds: Day of Starting, Volume, Frequency, Increase

### 6.1. Suggestion

In babies weighing <1 kg at birth, start nutritional feeds at 15–20 mL/kg/day and increase by 15–20 mL/kg/day. If the feeds are tolerated for around 2–3 days, consider increasing faster. For babies weighing ≥1 kg at birth, start nutritional feeds at 30 mL/kg/day and increase by 30 mL/kg/day.

### 6.2. Rationale

A Cochrane review (four RCTs, 588 subjects) compared slow daily increments (ranging from 15 to 20 mL/kg/day) *versus* fast daily increments of enteral feeding volume (ranging from 30 to 35 mL/kg/day) (LOE 1a) [14]. Fast increment did not increase the risk of NEC (pooled RR 0.97 (95% CI 0.54, 1.74)), mortality (pooled RR 1.41 (95% CI 0.81, 2.74)), or interruption of feeds (pooled RR 1.29 (95% CI 0.90, 1.85)). The trials individually reported that the fast daily increment group regained birth weight and reached full feeds faster (LOE 1b and 2b). As there was no subgroup analysis of ELBW babies, we suggest starting with a lower feed volume in ELBW babies—as in the control arm (15–20 mL/kg/day)—until more studies are available.

## 7. Type of Milk for Starting Feeds

### 7.1. Suggestion

The first choice is own mother’s expressed breast milk or colostrum. This should preferably be fresh; if not, provide previously frozen milk in the same sequence in which it was expressed.

Second choice: donor human milk.

Third choice: preterm formula.

### 7.2. Rationale

Freshly expressed human milk has numerous benefits for preterm babies [15]. Although there is no direct evidence comparing fresh *versus* frozen mother’s milk, the use of fresh milk makes sense because of the depletion of commensals, immune cells, immune factors, and enzyme activity that occurs with freezing. Neonates who receive an exclusively human milk-based diet (mother’s milk or donor human milk with human milk-based fortifier) have significantly lower rates of NEC compared to those who receive preterm formula or human milk with a bovine milk-based fortifier (LOE 1b) [16]. In another RCT, preterm infants who received an exclusively human milk diet (donor human milk and human milk-based human milk fortifier) had a lower incidence of NEC (21% *versus* 3%, *p* = 0.08) and surgical NEC (*p* = 0.04) compared to infants who received bovine milk-based preterm formula [17]. The use of donor human milk (while continuing bovine milk-based fortifier) *versus* preterm formula as a substitute for mother’s own milk does not reduce the rates of NEC [18]. The prohibitively high cost of human milk-based human milk fortifier is often quoted as an obstacle to using an exclusively human milk diet; however, a cost-effectiveness analysis showed that use of exclusively human milk-based products resulted in shorter duration of hospitalization (less by an average of 3.9 days in neonatal intensive care unit (NICU)) and savings of $8167 per extremely premature infant (*p* < 0.0001) because of the reduction in NEC [19].

## 8. Feeding Small for Gestational Age (SGA) Babies with/without History of Absent/Reversed End Diastolic Umbilical Flow (AREDF)

### 8.1. Suggestion

If the abdominal examination is normal, start feeding within 24 h of life, but advance slowly with volumes at the lowest end of the range. Advance feeds extremely slowly in the first 10 days among preterm SGA babies with gestation <29 weeks and AREDF. Make every effort to feed human milk, especially in SGA babies with AREDF and gestation <29 weeks.

### 8.2. Rationale

Mihatsch *et al.* [20] fed 124 VLBWI (35 had intra-uterine growth retardation (IUGR)) with a standardized protocol (LOE 2b). There was no statistical difference in the age to reach full feeds in the IUGR and non-IUGR groups (*p* = 0.6). In a multiple regression model, increased umbilical artery resistance, brain sparing, Apgar scores, umbilical artery pH, and IUGR did not predict the age to reach full feeds. In an RCT on SGA preterm babies (gestation of 27–34 weeks) who had abnormal antenatal umbilical Doppler flows, the incidence of NEC and feeding intolerance was not significantly different (*p* = 0.35 and *p* = 0.53, respectively) between the early feeders (*n* = 42; median age 2 days) and delayed feeders (*n* = 42; 7 days) (LOE 2b) [21].

In an RCT on preterm SGA infants, comparing minimal enteral feeding and no enteral feeding for five days, there was no difference in the rate of NEC (*p* = 0.76) and there was a trend towards shorter NICU stay in the enteral feeding group (*p* = 0.2) (LOE 2b) [22].

In the Abnormal Doppler Enteral Prescription Trial (ADEPT) RCT, 402 preterm SGA infants (<35 weeks gestation, birth weight < 10th centile) with absent or reversed end diastolic umbilical blood flow and cerebral redistribution were allocated to early or late onset of enteral feeding (Day 2 or 6, respectively) (LOE 1b) [23].The early feeding group reached full enteral feeds faster than the late feeding group (median (IQR) days: 18 (15–24) *versus* 21 (19–27), respectively; *p* = 0.003). There was no difference in the incidence of all-stage NEC (18% *versus* 15%, respectively; *p* = 0.42) and stage II–III NEC. Infants in the early feeding group had a significantly shorter duration of total parenteral nutrition (median difference 3 days, *p* < 0.001), a shorter duration of high dependency care (*p* = 0.002), and a lower incidence of cholestasis (*p* = 0.02). Eighty-six (21%) infants in this trial were below 29 weeks of gestation. The statistical test of interaction between treatment group and gestational age group (<29 weeks *versus* ≥29 weeks) was non-significant for age to reach full feeds (*p* = 0.38) and incidence of all stage NEC (*p* = 0.47), suggesting that the treatment effect was consistent across subgroups. The investigators published additional analysis from the ADEPT trial comparing infants of <29 weeks and ≥29 weeks of gestation [24]. The former group took significantly longer to reach full feeds compared to the latter (median age 28 days (Inter-quartile range (IQR) 22–40) *versus* 19 days (IQR 17–23), respectively; hazard ratio 0.35 (95% CI 0.3, 0.5)) and had a significantly higher incidence of NEC (39% *versus* 10%, respectively; RR 3.7 (95% CI 2.4, 5.7)). Infants <29 weeks in this trial tolerated very little milk in the first 10 days. Exclusive human milk feeding was the only protective factor.

## 9. Feeding Babies on Non-Invasive Ventilation

### 9.1. Suggestion

Increase feeds cautiously. Do not rely on abdominal distension as a sign of feeding intolerance, especially in babies weighing <1000 g.

### 9.2. Rationale

Non-invasive ventilation can cause abdominal distension, and nasal continuous positive airway pressure (nCPAP) decreases pre-and post-prandial intestinal blood flow in preterm infants (LOE 4) [25]. Jaile *et al.* [26] compared 25 premature infants on nCPAP with 29 premature infants not on CPAP (LOE 2b). Gaseous bowel distension due to CPAP developed in 83% of infants below 1000 g *versus* 14% of those weighing ≥1000 g. No cases of NEC were reported in the study; however, the sample size was too small to draw conclusions about NEC.

## 10. Feeding Babies with Systemic Arterial Hypotension

### 10.1. Suggestion

There is not enough evidence to make a suggestion.

### 10.2. Rationale

There is no published literature on feeding policies during systemic arterial hypotension.

## 11. Feeding Babies on Indomethacin or Ibuprofen

### 11.1. Suggestion

If the neonate is already on minimal feeds, continue to give trophic feeds until the indomethacin course finishes. If the neonate is fasting, introduce trophic feeds with human milk as per Section 3.

While there are no RCTs comparing feeding during indomethacin therapy *versus* ibuprofen, indirect evidence suggests ibuprofen may be the safer of the two.

### 11.2. Rationale

In the Ductus Arteriosus Feed or Fast with Indomethacin or Ibuprofen (DAFFII) trial, 117 infants (26.3 ± 1.9 weeks) who were on ≤60 mL/kg/day feeds and required treatment for patent ductus arteriosus (PDA) (75% to 80% received indomethacin) were randomized at 6.5 ± 3.9 days to receive trophic feeds or no feeds during the drug administration period [27]. Infants randomized to the trophic feeding subsequently required fewer days to reach 120 mL/kg/day (10.3 ± 6.6 days *vs*. 13.1 ± 7.8 days, *p* < 0.05). There is one retrospective study on 64 preterm infants (<29 weeks of gestation), half of whom had received indomethacin for PDA (LOE 4) [28]. There were no differences between the groups regarding feeding volumes, NEC incidence, or gastric residuals up to Day 7.

Ibuprofen is safer than indomethacin as it does not reduce mesenteric blood flow [29]. In a meta-analysis of 19 studies (956 infants), NEC rates were lower in the Ibuprofen group (typical RR 0.68 (95% CI 0.47, 0.99)) (LOE 1a) [30].

## 12. Assessment of Feed Tolerance

### 12.1. Suggestion

Do not check gastric residuals routinely. Check pre-feed gastric residual volume (GRV) only after a minimum feed volume (per feed) is attained. We suggest the following thresholds: <500 g: 2 mL, 500–749 g: 3 mL, 750–1000 g: 4 mL, >1000 g: 5 mL.

Do not check abdominal girth routinely.

Isolated green or yellow residuals are unimportant. Vomiting bile may indicate an intestinal obstruction or ileus. Withhold feeds in case of hemorrhagic residuals, as hemorrhagic residuals are significant.

### 12.2. Rationale

GRV is not as important a predictor of NEC as earlier thought. Below a certain feed volume, there is no point in checking the GRV. Among preterm babies on TPN, the mean + 2 SD value for GRV is about 4 mL (LOE 4) [31]. Mihatsch *et al.* [32] tolerated GRV up to 2 mL in infants <750 g and up to 3 mL in infants >750 g to 999 g. In a multiple regression model, the mean GRV and green residuals had no relationship with enteral feeding volume achieved by Day 14 (LOE 2b). In recent studies, ≤5 mL/kg has been used as a criterion for permissible GRV, but there are no comparisons between different cutoff values [33]. In a prospective study on 50 preterm infants, there was no correlation between feeding outcomes and GRV (mL/day) (LOE 2b) [34]. In a pilot RCT, 61 infants (24–32 weeks of gestation) were randomly allocated to receive routine evaluation of gastric residuals *versus* no routine evaluation [35]. There was no difference between the groups regarding volume of feeds at 3 weeks of age, growth, and days on TPN. Infants without routine evaluation of gastric residuals reached full feeds six days earlier.

In a case-control study on VLBWI with (*n* = 17) and without (*n* = 17) NEC, the mean maximum GRV as a percentage of the previous feed was 113% among subjects with NEC and 43% in controls (LOE 4) [36]. Hemorrhagic residuals, but not green residuals, were associated with NEC.

A study that suggested a relationship between modest GRV and NEC was a case-control study on 51 VLBWI with proven NEC and 102 healthy controls (LOE 4) [37]. The study was criticized for its choice of controls. There was a difference in the maximum GRV as a percentage of the corresponding feed volume between the NEC group and the controls (median (IQR): 40 (24, 61) *vs*. 14 (4, 33), *p* < 0.001), but with a large overlap between groups.

Although most studies have downplayed the importance of GRV, two retrospective studies, mentioned above, suggest there could be a relationship with NEC.

Green residuals could be due to duodenogastric reflux or overzealous aspiration, which could suck back duodenal contents (LOE 5) [38,39]. Studies have not found an association between green residuals and NEC (LOE 2b and 4) [32,36].

Abdominal girth is not a reliable measure of feed tolerance. There is a paucity of studies evaluating an increase in girth with clinical outcomes. It is highly prone to intra- and inter-observer variation. Abdominal circumference may vary by 3.5 cm during one feeding cycle in normal premature infants (LOE 4) [40]. It correlates with time from last defecation (*p* = 0.0001). Among term infants, the mean inter-observer difference is up to 1 cm [41].

## 13. Management of Residuals

### 13.1. Suggestion

Push back GRV of up to 5 mL/kg or 50% (whichever is higher) of the previous feed volume. If it recurs, subtract the residual volume from the current feed.

If the GRV is >5 mL/kg and >50% of the previous feed volume, push back the GRV up to 50% of the feed volume and do not give the current feed. If this happens again, consider slow bolus feeds or withholding feeds, depending on the clinical condition.

If the problem of residual volumes persists despite slow bolus feeds, consider decreasing the feed volume to the last well-tolerated feed volume.

Use the smallest volume syringe for checking residuals. Take care to aspirate gently.

After a feed, nurse the baby in the prone position for half an hour.

### 13.2. Rationale

The rationale for 5 mL/kg is covered in Section 11. The criterion of 50% is a round figure approximately equal to the cutoff from the study by Cobb *et al.* [37]. Pushing back partially digested gastric aspirates may replenish acid and enzymes that aid in the digestive process [42].

There is a paucity of data regarding the role of slow bolus feeding. In a physiologic study on pre-terms comparing a 120-min infusion of feeds compared to bolus feeds, the former was associated with faster gastric emptying, lower GRV, and more frequent duodenal motor responses (LOE 2b) [43]. Whether these theoretical advantages of slow bolus translate into clinical benefits is unclear, but there is a physiological basis for trying. In a Cochrane meta-analysis comparing continuous nasogastric *versus* intermittent bolus feeding in VLBWI, the continuous method resulted in a longer time to reach full enteral feeding (weighted mean difference (WMD) 3 days (95% CI 0.7, 5.2)), with no difference in growth or incidence of NEC (LOE 1a−) [44].

The narrower the diameter of the syringe, the less pressure is applied while pulling (as opposed to pushing) (LOE 4) [45]. Hence, smaller volume syringes are preferred.

In an RCT, the decrease in the volume of gastric residuals was lower in the prone position than in supine, and the rate of decrease of gastric residual volume was highest in the first half hour after the feed [46].

## 14. Clinical Diagnosis of Gastro-Esophageal Reflux (GER)

### 14.1. Suggestion

Do not rely on apnea, desaturation, or bradycardia; or behavioral signs, such as gagging, coughing, arching, and irritability, as signs of GER in preterm babies.

### 14.2. Rationale

The relationship between GER and cardio-respiratory events is controversial. Early studies either used a pH probe, which is unable to detect non-acid reflux; or used only the multi-channel intraluminal impedance (MII) probe, which underestimates acid GER events. The modality of choice is combined MII-pH monitoring.

In MII-pH studies on 71 preterm infants (mean birth weight 1319 g) there were 12,957 cardiorespiratory events and 4164 GER episodes, but GER preceded less than 3% of all cardiorespiratory events (LOE 2b) [47].

In another MII study on 19 preterm infants, the frequency of apneas occurring within 20 s of reflux episodes was not significantly different from that during reflux-free periods (LOE 2b) [48].

In a 24-h pH-MII study on 21 healthy premature infants, only 25% of reflux events were acidic [49]. Episodes that reached the proximal esophagus were also unassociated with cardio-respiratory events.

Contrary to the above, a group from Italy has published two reports using pH-MII monitoring that support the relationship between GER and apnea ≥5 s (LOE 2b) [50,51]. The frequency of apnea in the 30 s after GER was greater than in the 30 s before GER (*p* = 0.01). Apnea was shown to be associated with non-acid MII-GER episodes (*p* = 0.000), but not with acid GER episodes (*p* = 0.137).

We conclude that GER is probably not associated with cardio-respiratory events. At worst, it may cause brief episodes of apnea, and these are primarily from non-acidic episodes.

Snel *et al.* [52] studied 14 preterm infants (gestation of 26–35 weeks) who underwent continuous esophageal pH monitoring and videography (LOE 4). For each episode of acid GER, a 10-min video recording was analyzed and compared with a 10-min clipping when the esophageal pH exceeded 4. The recordings were randomized and viewed independently by two masked observers. There was no relationship between behavioral cues and reflux. The association of behavioral patterns with GER has only been reported in a study on eight term babies [53].

There is no difference in behavioral symptom scores after treatment with cisapride or omeprazole, emphasizing that they are possibly not because of GER (LOE 2b) [54,55].

## 15. Body Position for Treatment of GER

### 15.1. Suggestion

Place the baby in the left lateral position after a feed and turn over to the prone position about half an hour later. Elevate the head end to 30°. Place the infant supine for sleeping at home.

### 15.2. Rationale

Among 22 preterm infants with regurgitation who underwent 24-h recording of pH-MII in four body positions, the left lateral position showed the lowest esophageal acid exposure in the early post-prandial period and the prone position in the late post-prandial period (LOE 2b) [56].

Preterm infants are not an exception to the supine sleep recommendation for home care, because of the increased risk of sudden infant death syndrome (SIDS) among preterm infants [57,58].

## 16. Medications for Treatment of GER

### 16.1. Suggestion

Do not use domperidone, H2-blockers, or proton-pump inhibitors for the treatment of GER.

### 16.2. Rationale

In the only study evaluating domperidone, 13 infants with suspected GER treated with domperidone were compared to 13 untreated controls with suspected GER (LOE 2b) [59]. On 24-h pH-MII monitoring, the frequency of GER episodes was higher in the domperidone group (*p* = 0.001). A crossover trial on 18 infants comparing metoclopramide with a placebo had similar findings [60]. Domperidone is associated with prolongation of QTc interval in neonates above 32 weeks of gestation [61]. QTc prolongation has not been demonstrated in more premature infants (*n* = 40); however, we need additional data to declare domperidone as safe [62].

Ranitidine is associated with a higher incidence of late onset sepsis (LOE 4) [63] and NEC in preterm neonates (LOE 3b) [64]. In a study on VLBWI comparing *n* = 91 who had received ranitidine with *n* = 183 who had not, the odds of developing sepsis in the ranitidine group was 5.5-fold higher and of NEC 6.6-fold higher (LOE 2b) [65]. Mortality was also higher in neonates receiving ranitidine (*p* = 0.003).

In a double-blind placebo-controlled crossover study in preterm neonates, omeprazole reduced intra-gastric acidity but not the frequency of reflux symptoms (L4E 2b) [66]. In another double-blind RCT on 52 premature neonates with suspected GER, there were no differences between the esomeprazole and placebo groups with respect to percentage change from baseline in total number of signs and symptoms related to GER and number of reflux episodes [67].

The association of GER and cardio-respiratory events is itself questionable. The only study that shows an association concluded that it is with the non-acid reflux [51]. Observational data suggests that gastric acid suppression is associated with serious adverse events. Hence, there is little justification for pharmacological gastric acid suppression in the treatment of GER.

## 17. Thickening Feeds for GER

### 17.1. Suggestion

Avoid thickeners in the treatment of presumed GER.

### 17.2. Rationale

There are no RCTs evaluating the thickening of feeds in an exclusively neonatal population [68]. One non-randomized trial compared smectite in neonates with GER measured by 24-h pH monitoring (LOE 4) [69]. The use of open-label thickeners contaminated the groups. Postural therapy combined with smectite was followed by a decrease in GER (*p* < 0.05). In another non-randomized trial on 24 infants, a formula thickened with amylopectin did not decrease the incidence of GER-related apneas or apnea of prematurity [70]. There is no evidence to support the use of rice cereal or thickened formulae.

The safety of thickeners in preterm neonates is questionable. Xanthan gum-based, carob bean gum-based, and pectin-based thickeners have all been reported to cause NEC (LOE 4) [71,72,73].

## 18. Feed Duration and Route of Feeding for Treatment of GER

### 18.1. Suggestion

If one strongly suspects GER, and re-positioning does not help, one may increase the feed duration to 30–90 min. Make all attempts to reduce the duration back to a shorter duration as soon as possible. Use continuous or trans-pyloric feeding as a last resort for the management of GER and avoid them as far as possible. There is still insufficient evidence to recommend the use of erythromycin for the prevention or treatment of feed intolerance.

### 18.2. Rationale

The association of cardio-respiratory events with GER is itself questionable. Hence, the evaluation of any intervention to “treat” GER is fraught with problems. Altering feed duration and body position is probably less harmful than medications for GER.

The physiological benefits of slow gavage feeds have been described earlier (Section 12) [43]. However, in a cross-over RCT on 30 preterm infants who received 21 ± 1.5 mL/kg of milk per feed using bolus gavage feeding over 10 min or slow gavage feeding over 1 h, there was no difference in terms of frequency of apneas (>4 s), bradycardias, and desaturations (LOE 2b) [74]. The Cochrane meta-analysis comparing continuous nasogastric milk feeding *versus* intermittent bolus feeding in VLBWI has been described earlier (Section 12) [44].

In view of the above information, there may be limited justification for trying out slow enteral feeding but no justification for prolonged slow gavage feeds or continuous feeds.

A Cochrane review on nine RCTs of transpyloric *versus* gastric feeding in preterm infants concluded that transpyloric feeding did not improve feed tolerance or growth and had an increased risk for cessation of feeds and mortality (LOE 1a) [75]. Moreover, this review is not directly applicable because the trials were on transpyloric enteral feeding as an initial feeding strategy, rather than as treatment for GER.

In a retrospective study on 72 VLBWI with apnea/bradycardia due to presumed GER, the authors observed a reduction in the average number of apnea/bradycardia episodes with transpyloric feeding (*p* = 0.02) (LOE 4) [76].

A Cochrane review on erythromycin (three prevention and seven treatment RCTs until 2007) included studies that varied in definition of feed intolerance and in the measurement, analysis, and reporting of outcomes [77]. Therefore, meta-analysis could not be done. Subsequently, in a placebo-controlled RCT on high-dose oral erythromycin (50 mg/kg/day), there was no significant difference reported in the time to reach full feeds [78]. Another RCT on the efficacy of intermediate-dose erythromycin in 45 VLBW infants >14 days of age with feed intolerance reported a significantly lower number of days to achieve full feeds (36.5 ± 7.4 days *versus* 54.7 ± 23.3 days; *p* = 0.01) and a lower number of days on TPN in the erythromycin group [79].

## 19. Prevention of Nutrient Loss during Slow Gavage or Continuous Feeds

### 19.1. Suggestion

Use the shortest possible extension tubing from the syringe to the baby to prevent nutrient loss. Do not draw up extra milk—other than that used for priming—into the syringe. If slow bolus feeds have to be used, keep the duration to a minimum.

### 19.2. Rationale

In an experimental study, continuous feeding resulted in (mean) 40% loss of fat, 33% of calcium, and 20% of phosphorus (LOE 2b) [80]. Infusion via gravity resulted in 6%, 9%, and 7% losses, respectively. Infusion by pump over 30 min resulted in intermediate losses. Fat adheres to the inner wall of the tubing, resulting in losses.

## 20. Human Milk Fortification

### 20.1. Suggestion

Start fortification when enteral intake reaches 100 mL/kg/day. Start at a concentration of 1:50 and if this is tolerated for 48 h increase to 1:25.

### 20.2. Rationale

Most authors have started human milk fortification at 100 mL/kg/day and at a concentration of 1:50 (LOE 5) [81,82,83]. In the only RCT that compared starting fortification with a human milk-based fortifier at 100 mL/kg/day *versus* starting at 40 mL/kg/day, there was no difference in outcomes (LOE 1b) [16]. However, it is unclear whether this can be extrapolated to a bovine milk-based fortifier. In a retrospective study on infants <31 weeks of gestation, the authors compared 53 infants who received HMF from the first feed with *n* = 42 who received it after reaching a feed volume of 50–100 mL/kg/day [84]. There were no differences in weight gain, but the early fortification group had a lower incidence of elevated alkaline phosphatase levels. Thus, more RCTs are required before recommending fortification at less than 100 mL/kg/day or an initial concentration less than 1:50.

## 21. Glycerin Enemas to Promote Feed Tolerance

### 21.1. Suggestion

Do not use daily glycerin suppositories to reduce the time to full enteral feeding. If one uses a trial of glycerin tips on a case-by-case basis, one should take into account the normal stooling pattern in preterm infants and volume of milk ingested to decide on the need for a glycerin tip.

### 21.2. Rationale

There is a relationship between gestation and the time of passage of the first stool: the lower the gestation, the longer the time taken [85].

In a study on 41 ELBW infants, there was an inverse correlation between feed volume on Day 14 and the last day of passing meconium but there was no correlation with the first day of passing (LOE 2b) [86]. In an observational study involving historical controls, VLBWI undergoing meconium evacuation by routine glycerin enema starting from Day 1 of life achieved full enteral feeding faster than controls (hazard ratio 2.9; 95% CI 1.8, 4.8) (LOE 2b) [87]. A subsequent RCT showed that daily glycerin suppositories did not reduce the time to full enteral feeding in infants born at less than 32 weeks of gestation (LOE 1b) [88]. Glycerin enemas may also cause rectal tears.

## 22. Conclusions

We suggest that physicians taking care of VLBW infants should aim to reach full feeds by about 2 weeks of age in neonates weighing <1000 g at birth and by about one week in neonates weighing 1000–1500 g at birth. A 3-hourly feeding regimen can be introduced for infants weighing >1250 g. Trophic feeds (10–15 mL/kg/day) should be started preferably within 24 h of life, but caution should be exercised in extremely preterm, ELBW or growth restricted infants. For babies weighing ≥1 kg at birth, we suggest starting nutritional feeds at 30 mL/kg/day and increase by 30 mL/kg/day. The first choice of milk is mother’s own fresh milk. Among SGA infants with AREDF and a normal abdominal examination, feeding can be started within 24 h of life, and advanced cautiously. Feeds should be advanced cautiously in VLBW infants on non-invasive ventilation. If the neonate is already on minimal feeds, trophic feeds should be continued until the indomethacin course finishes. We advise against checking gastric residuals routinely. Abdominal girth should not be checked routinely. Isolated green or yellow residuals are unimportant. We suggest that GRV of 5 mL/kg or 50% (whichever is higher) of the previous feed volume should be pushed back and the amount subtracted from the current feed. One should not rely on apnea, desaturation or bradicardia; or behavioural signs as markers of GER in preterm babies. Body positioning in the left lateral or prone position may help with GER but medications and thickeners are not recommended. Slow gavage feeding may be attempted in GER after taking care to prevent nutrient losses. Human milk may be fortified after enteral intake of 100 mL/kg/day is reached. Glycerin enemas should not be used with the intention of reducing the time to full enteral feeding.
